# Structure and Tribological Properties of TiN/DLC, CrN/DLC, TiAlCN/DLC, AlTiCN/DLC and AlCrTiN/DLC Hybrid Coatings on Tool Steel

**DOI:** 10.3390/ma18174188

**Published:** 2025-09-06

**Authors:** Marcin Staszuk, Daniel Pakuła, Magdalena Olszowska, Anna Kloc-Ptaszna, Magdalena Szindler, Andrzej N. Wieczorek, Rafał Honysz, Ewa Jonda, Marcin Basiaga

**Affiliations:** 1 Faculty of Mechanical Engineering, Silesian University of Technology, Konarskiego 18A, 44-100 Gliwice, Poland; daniel.pakula@polsl.pl (D.P.); magdols407@student.polsl.pl (M.O.); anna.kloc@polsl.pl (A.K.-P.); magdalena.szindler@polsl.pl (M.S.); rafal.honysz@polsl.pl (R.H.); ewa.jonda@polsl.pl (E.J.); 2 Faculty of Mining, Safety Engineering and Automation Industry, Silesian University of Technology, Akademicka 2A, 44-100 Gliwice, Poland; andrzej.n.wieczorek@polsl.pl; 3 Faculty of Biomedical Engineering, Silesian University of Technology, Roosevelta 40, 41-800 Zabrze, Poland; marcin.basiaga@polsl.pl

**Keywords:** PVD, DLC, anti-wear coatings, tribology, SEM, AFM, scratch test

## Abstract

In view of the need to increase the durability of working tools exposed to intense friction, this study analysed hybrid coatings (TiAlCN, AlTiCN, AlCrTiN, TiN, CrN) with a DLC (Diamond-Like Carbon) layer, deposited using PVD (Physical Vapour Deposition) methods (arc evaporation and magnetron sputtering). The structural characteristics of the coatings were determined using SEM (Scanning Electron Microscope) and AFM (Atomic Force Microscope) microscopy, as well as Raman spectroscopy, which confirmed the compact structure and amorphous nature of the DLC layer. Tribological tests were performed using a ball-on-disc test, revealing that DLC hybrid coatings significantly reduce the coefficient of friction (stabilisation in the range of 0.10 to 0.14 due to DLC graphitisation), limiting tool wear even under increased load. The SEM-EDS (Scanning Electron Microscope-Energy Dispersive Spectroscopy) microscopic examination revealed that the dominant wear mechanisms are abrasive and adhesive damage, and the AlCrTiN/DLC system is characterised by low wear and high adhesion (L_c_ = 10^5^ N), making it the optimal configuration for the given loads. Microhardness tests showed that high hardness does not always automatically translate into increased wear resistance (e.g., the AlTiCN coating with 4220 HV showed the highest wear), while coating systems with moderate hardness (TiAlCN/DLC, CrN/DLC) achieved very low wear values (~0.17 × 10^−5^ mm^3^/Nm), which highlights the importance of synergy between the hardness of the sublayer and the low friction of DLC in the design of protective coatings.

## 1. Introduction

In recent years, there has been a dynamic increase in production in many sectors of the economy, including the metalworking industry, which supplies components for various industries. In the face of increasing costs and the energy crisis, greater emphasis is being placed on extending the service life of tools, which is often associated with the use of lubricants. However, increasing environmental awareness and tightening regulations on sustainable industry encourage the search for alternative solutions. One of them is self-lubricating coatings, such as diamond-like carbon (DLC), which effectively reduce the coefficient of friction.

Despite the numerous advantages, DLC coatings are characterised by relatively low adhesion to the substrate, which limits their durability. As the authors report in [1], the problem of poor adhesion is attributed to two main reasons. The first reason is that carbon diffuses into the iron network, leading to the formation of a graphite layer at the interface, which weakens the bonding strength at the interface of these phases. The second reason is the high level of internal stresses, which results mainly from the large difference in the thermal expansion coefficient between the ta-C (tetrahedral amorphous Carbon) coating and the stainless steel substrates (at room temperature αDLC ≈ 2.3 × 10^−6^/K and αsteel ≈ 11.7 × 10^−6^/K). High internal stresses are a characteristic of both anhydrous ta-C and hydrogenated DLC (a-C:H) coatings, which belong to the broad family of amorphous coatings. The stress level of hydrogenated coatings depends on their hydrogen content, the energy of the particles during deposition, and the process conditions, all of which affect their structure and mechanical properties. Excessive stresses, both compressive and tensile, can lead to cracking, peeling, or weakened adhesion of the coating to the substrate. Therefore, controlling the deposition parameters and using transition layers are key to improving the durability and stability of DLC coatings, regardless of their chemical composition. Research on improving the adhesion of DLC coatings indicates two promising solutions. The first solution consists of the use of a transition layer between the substrate and the DLC coating. Transition metals such as Cr, Ni, Ti, Al, W, and Zr can be doped into DLC during the coating growth process or can be deposited on the substrate as an intermediate layer to reduce the internal stresses of the coating and improve its adhesion. Titanium has a high affinity for carbon and good compatibility with steel [1,2,3,4,5,6].

In addition, the thermal expansion of Ti is 8.6 × 10^−6^/K, which can compensate for the large thermal expansion mismatch between the DLC layers and the steel substrates, which in turn leads to a reduction in internal stresses. On the other hand, chromium, due to its chemical characteristics, can cause carbon diffusion into the substrate and ensure the formation of a carbide phase, which in effect increases the adhesion of diamond layers to the substrate [1,7,8]. The second solution involves the use of hybrid coating technology, which combines different deposition processes [9,10,11,12]. The main methods for producing diamond-shaped layers include physical and chemical vapour deposition. In the case of DLC coating deposition, physical deposition technology currently prevails over the chemical method. This is mainly due to the fact that chemical vapour deposition methods are not considered environmentally friendly. However, it is worth noting that CVD (Chemical Vapour Deposition) methods, despite environmental limitations, enable the production of hydrogenated coatings (a-C:H (amorphous hydrogenated Carbon)), in which the presence of hydrogen comes from hydrocarbon ions, such as acetylene. Although these types of coatings are less useful in cutting tools due to their lower hardness, they have very good tribological properties in sliding applications. Physical PVD methods, such as cathodic arc evaporation or sputtering, allow the production of anhydrous and harder DLC coatings, e.g., ta-C, which are more suitable for heavy-duty applications. Thus, the deposition method determines not only the environmental impact but also the final performance properties of the coating. Among the PVD (Physical Vapour Deposition) methods, the most frequently used methods for diamond-like coatings are sputtering and arc evaporation. It should be noted that the coating deposition method is one of the factors that influences its final properties. In the case of hard DLC coatings with a predominant sp^3^ fraction, methods that generate large amounts of energy are used, e.g., arc processes. In the case of coatings with a predominant sp^2^ fraction, processes involving ion sputtering, for example magnetron sputtering or ion beam deposition, are completely sufficient to achieve the intended structure. The fundamental factor controlling the ratio of sp^2^ to sp^3^ bonds in DLC coatings is the energy of ions that bombard the substrate during the deposition process. Methods that generate higher energy, such as cathodic arc or magnetron sputtering with additional ion irradiation, promote an increase in the sp^3^ bond content, resulting in harder and more resistant coatings. On the other hand, lower ion energy, characteristic of some CVD techniques, leads to the formation of structures with a higher sp^2^ content, resulting in more flexible and softer coatings with favourable tribological properties in sliding applications. Therefore, selection of the deposition method and process parameters is crucial for obtaining optimal coating properties tailored to specific applications [13,14,15,16,17,18,19,20,21].

Much effort has been put into the development of PVD processes that would enable the increase in the density of ions and atoms of the coating material reaching the substrate surface during deposition. Achievement of this goal was made possible by the use of appropriately shaped magnetic fields in the magnetron sputtering method. This method has several significant advantages over other vacuum coating techniques, which has led to the development of a large number of commercial applications, from microelectronic production to simple decorative coatings. The numerous advantages of magnetron sputtering include [9] a high deposition rate, ease of sputtering of any metal, alloy, or compound, high purity of the layers obtained, very high adhesion, excellent coverage of steps and small elements, the possibility of coating heat-sensitive substrates, ease of automation, and excellent uniformity on large-area substrates, for example architectural glass [22,23,24].

In connection with this, studies have been conducted on the structure and mechanical properties of a wide range of hybrid coatings, TiAlCN/DLC AlTiCN/DLC, AlCrTiN/DLC, TiN/DLC and CrN/DLC, deposited on tool steel by arc evaporation and magnetron sputtering. The first layer provides good adhesion and resistance to loads, with TiAlCN, AlTiCN, AlCrTiN, TiN, and CrN acting as buffers that reduce stresses resulting from differences in thermal expansion and structural changes during coating deposition, and the DLC layer is responsible for low friction and wear resistance.

## 2. Material

The tested coatings were deposited on a W360-type steel substrate with the chemical composition shown in Table 1. Steel was hardened and tempered twice before coating. After heat treatment, the hardness of the steel was 57 ± 0.3 HRC (Hardness Rockwell C). Before coating, the substrates were ground on grinding wheels of decreasing gradation and polished using polishing cloths and diamond suspensions. The steel surfaces to be coated were sanded with decreasing grit size from 220 to 1200. The samples’ surfaces were then polished using diamond suspensions ranging from 9 um to 1 um. Between each grinding and polishing stage, the samples were rinsed with water and dried with compressed air. The substrates were rinsed with acetone in an ultrasonic cleaner and dried with a hot air blower. The samples were discs with a diameter of 20 mm and a height of approximately 6 mm.

On the prepared steel substrate, the TiAlCN, AlTiCN, AlCrTiN, TiN, and CrN coatings were first deposited by cathodic arc evaporation–physical vapour deposition (CAE-PVD), and then, in a separate process, DLC coatings with a chromium sublayer were deposited by magnetron sputtering–physical vapour deposition (MS-PVD). Table 2 lists the type of coatings to be tested and the thickness of the coatings tested. CAE-PVD/MS-PVD coatings can be called hybrid coatings because they were obtained by deposition in two separate PVD techniques. The investigated coatings were made using parameters previously optimised by the company with which cooperation was initiated in this field.

## 3. Methodology

The coating topography, chemical composition, and damage resulting from the tribological test were examined using a Zeiss Supra 35 scanning electron microscope (Zeiss, Oberkochen, Germany) equipped with a Thermo Scientific EDS detector (Thermo Fisher Scientific, Waltham, MA, USA). The applied accelerating voltage was in the range of 5–20 kV. The surfaces of the preparations after tribological testing were coated with gold to ensure electrical charge dissipation during SEM testing.

The morphology of the obtained coatings was also examined using a Park Systems XE-100 atomic force microscope (Park Systems XE-100, Suwon, Republic of Korea). Measurements were carried out in noncontact mode, examining areas of dimensions 1 µm × 1 µm. A PPP-CONTSCR-type cantilever was used (high resonance frequency > 200 kHz, high spring constant > 20 N/m).

The qualitative examination of the DLC coating was performed using a Renishaw in Via Reflex Raman Spectrometer (Renishaw GmbH, New Mills, UK), and the excitation wavelength was 514 nm; spectra were obtained in the range of 100 cm^−1^ to 3200 cm^−1^.

Wear resistance tests were performed using the CSM Instruments tribometer (Anton Paar, Peseux, Switzerland) using the “ball-on-disc” method at room temperature. The test was carried out in the reciprocating mode. A 5.55 mm diameter Al_2_O_3_ ball was used as a counter sample. The test parameters were as follows: load = 10 N, number of cycles = 20,000, amplitude = 6 mm, speed = 5.31 Hz. The friction coefficient was recorded as a function of the number of cycles during the test. Wear profiles were measured using a Tylor-Hubson Sutronic 25 contact profilometer (Taylor Hobson, Leicester, UK). The volume of the worn material was determined on the wear profile area and the length of the wear traces. Wear rate [17], defining the wear rate of the material due to abrasion, was calculated from the following relationship (1):

Wear rate = V/(F_s_) [mm^3^/Nm] (1)

where V—volume of worn material (mm^3^); F—contact force (N); s—friction path (m).

The adhesion of the coating to the substrate was measured using the Revetest RST scratch tester from Anton Paar (Anton Paar, Peseux, Switzerland). A progressive load of 0 to 200 N was applied and the measurement path length was 10 mm. The critical load L_c_, at which the adhesion of the coating was lost, was determined based on the acoustic emission value recorded during the measurement and observation of the scratches created during the test.

The nanoindentation test was performed using an open platform equipped with a CSM micro- and nanocombination tester (Anton Paar, Peseux, Switzerland). The test was carried out at five measurement points for each sample. A time-varying force was applied for the tests in the range of 0.1 mN to 75 mN with a step of 7.5 s.

## 4. Results and Discussion

**Figure 1:**
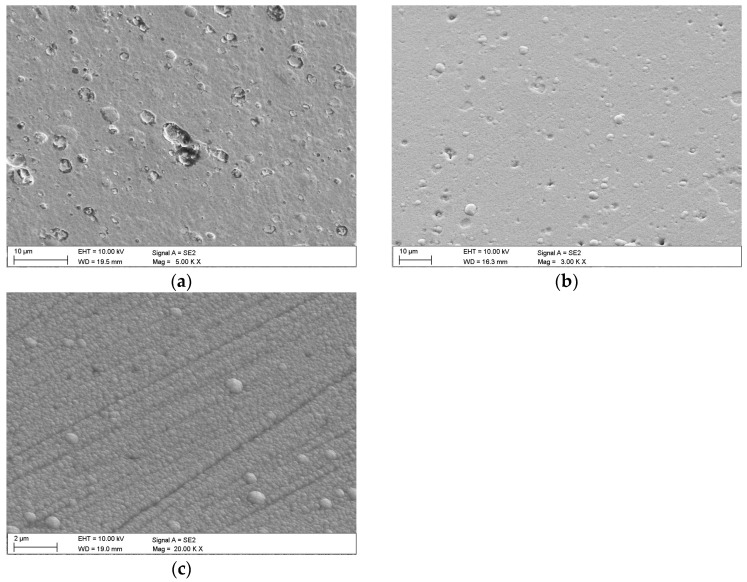
Topography of investigated coatings: (**a**) CrN, (**b**) CrN/DLC, (**c**) DLC.

Topographic studies have shown that the coatings deposited by the CAE-PVD method (Figure 1a) are characterised by a compact structure, but their surface contains a large number of irregular pores and droplets, which is typical for the arc deposition process. Craters resulting from the precipitation of larger droplets were also observed. Despite this, no microcracks or significant coating chipping was found. The CAE-PVD/DLC hybrid coatings (Figure 1b) are characterised by a clearly smoother structure, although drops and pores can still be seen in them. This is because the DLC layer covers the rough surface of the CAE-PVD coatings, not completely levelling its unevenness. In the case of the DLC coating, the surface is smooth and compact, free of drops and pores typical of coatings deposited by the arc method. This is consistent with the characteristics of the magnetron sputtering process (MS-PVD) (Figure 1c).

**Figure 2:**
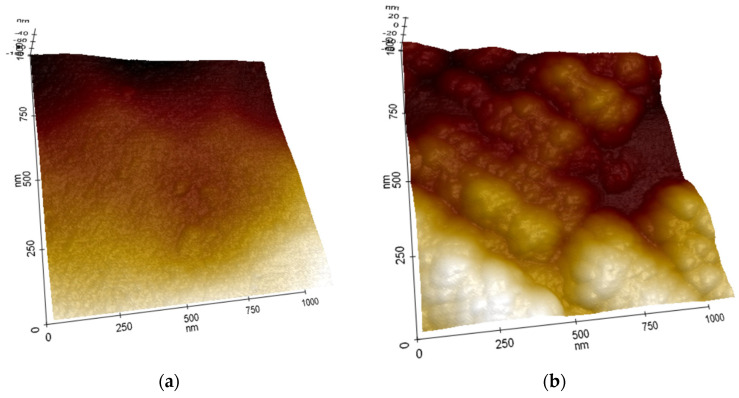
Morphology of investigated coatings: (**a**) TiN, (**b**) TiN/DLC.

Studies of the coating morphology using an atomic force microscope (AFM) showed that the CAE-PVD/DLC hybrid coatings (Figure 2) were characterised by a much more developed surface compared to coatings without a DLC layer, which was not visible in SEM studies. Furthermore, no scratches or imperfections resulting from substrate preparation were found on their surface, indicating a relatively large thickness of the deposited coatings.

**Figure 3:**
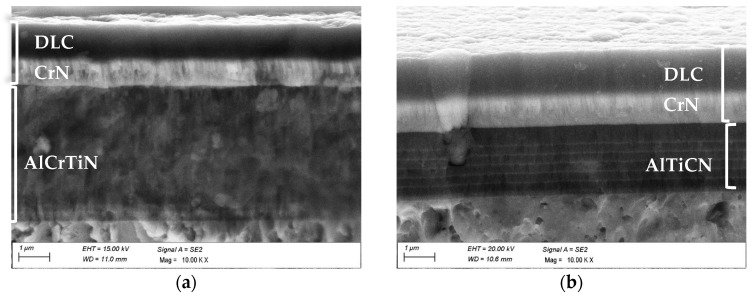
Microstructure of investigated coatings: (**a**) AlCrTiN/DLC, (**b**) AlTiCN/DLC.

Analysis of the fracture structure confirmed that the hybrid coatings exhibit a multilayer architecture (Figure 3). For the AlCrTiN/DLC, TiN/DLC, and CrN/DLC systems, the core layers (AlCrTiN, TiN, or CrN) were deposited using the CAE-PVD method, and each was overlaid with a DLC coating deposited by MS-PVD. In all cases, a CrN sublayer approximately 1 µm thick was positioned beneath the DLC layer, which itself reached a thickness of about 1.2 µm.

In contrast, the TiAlCN/DLC and AlTiCN/DLC systems incorporated multilayer TiAlCN or AlTiCN base structures. As with the single-layer coatings, these were subsequently covered with a DLC coating supported by a CrN sublayer. The layer thicknesses for all coatings are reported in Table 2. EDS microanalysis confirmed the chemical composition of each layer, while microstructural examination revealed a fine-grained morphology consistent with the T-zone in Thornton’s model [25].

**Figure 4:**
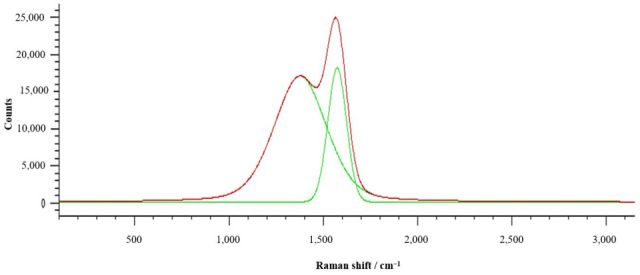
Raman spectrum for DLC coating.

On the basis of the Raman spectrometer tests, the presence of a diamond-like carbon (DLC) coating was confirmed by a characteristic spectrum pattern indicating the presence of an amorphous carbon phase (Figure 4). For the graphite structure, an intense signal around 1580 cm^−1^ is typical, while the diamond structure is revealed as a narrow band located at 1332 cm^−1^. In the case of intermediate materials, such as DLC coatings or amorphous carbon, the Raman spectrum takes the form of broad overlapping bands. Two characteristic features are observed:

- The D band (~1345 cm^−1^), which corresponds to the breathing modes of sp^2^ carbon rings and becomes Raman-active in the presence of disorder or finite crystallite size;
- The G band (~1560 cm^−1^), which originates from the stretching vibrations of sp^2^ carbon–carbon bonds in the hexagonal lattice.

The spectrum obtained for the DLC coating, presented in Figure 4, was fitted as a sum of two Gaussian functions corresponding to the D and G bands. Prior to deconvolution, the baseline was corrected by subtracting a linear background. The fitting was carried out using the area ratio (A_D/A_G) rather than the peak height ratio, since the integrated area provides a more accurate measure for broad and overlapping bands typically found in amorphous carbon materials. The visible broad and partially overlapping bands constitute a typical image of amorphous materials with mixed hybridisation, which clearly indicates the amorphous nature of the obtained layer and confirms its assignment to the DLC type.

**Figure 5:**
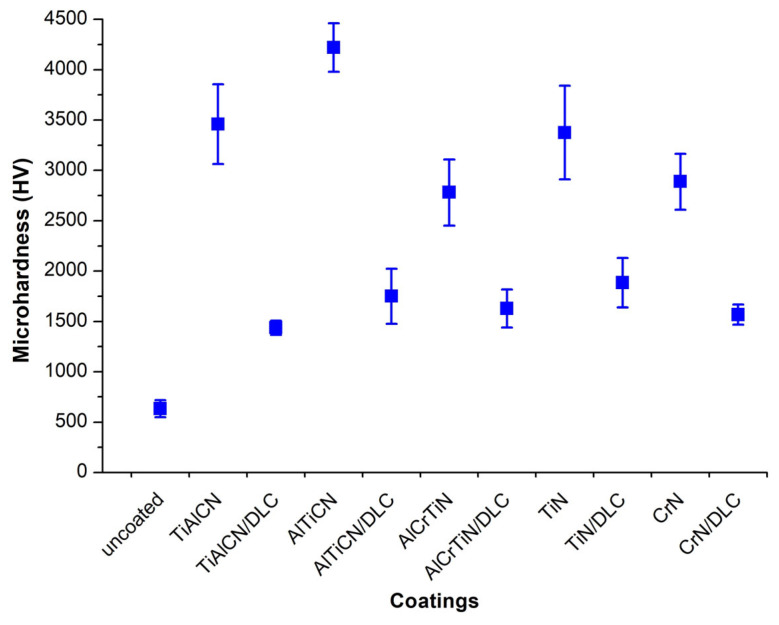
Microhardness of the surfaces of the tested materials.

The microhardness results (Figure 5) indicate that the highest microhardness among the coatings analysed was achieved by the AlTiCN coating (4220 HV). Similar high microhardness was also demonstrated by the TiAlCN coating (3458.78 HV) and TiN (3375 HV). Hybrid coatings of the MeN/DLC type (e.g., TiN/DLC—1884.4 HV) were characterised by significantly lower microhardness than their counterparts without a DLC layer, which can be explained by the relatively lower hardness of the DLC coating itself (1400 HV) and its influence on the characteristics of the entire coating system. At the same time, despite the lower microhardness, the presence of the DLC layer significantly improves tribological properties, as demonstrated, among others, in studies [26], where the DLC layer increases wear resistance while maintaining moderate hardness.

**Figure 6:**
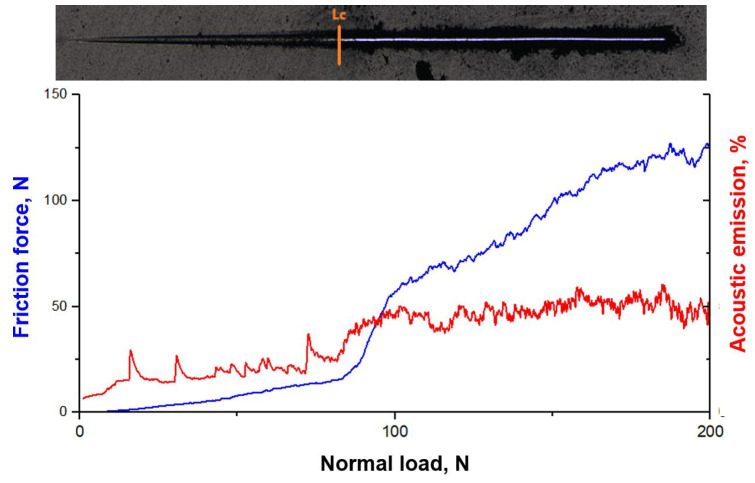
Relationship between the friction force and acoustic emission and the value of the normal load for the AlTiCN/DLC coating.

Based on adhesion tests using the scratch test method (Figure 6), the highest L_c_ values are shown by hard nitride coatings: TiAlCN (127 N), TiN (126 N), and AlCrTiN (120 N). Among hybrid coatings with a DLC layer, the best result was achieved by the TiN/DLC coating (124 N), whose strength is almost equal to the TiN coating without a DLC layer, indicating very good compatibility of both layers. The deposition of a DLC layer on the tested coatings in many cases affects the value of the critical load L_c_ in a differentiated way. In the case of CrN and AlTiCN coatings, the application of DLC improved the Lc value, which indicates a positive effect of DLC on the adhesion of these weaker base layers. In turn, for TiAlCN and AlCrTiN coatings, the application of a DLC layer reduced the critical load, which may result from internal stresses or non-optimal interfacial properties. The lowest scratch resistance is demonstrated by pure DLC (76 N), which confirms the necessity of using intermediate layers to increase its adhesion in practical applications (Table 3).

**Figure 7:**
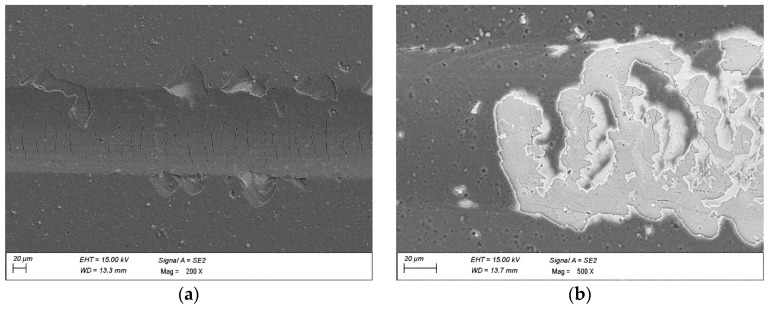
Damage after the scratch test in the coatings: (**a**) AlCrTiN, (**b**) AlCrTiN/DLC.

Scratch test results highlight the different failure mechanisms of single-layer and hybrid coatings (Figure 7). For CAE-PVD coatings, damage initiated near Lc with minor cracks and edge delamination, progressing to full detachment of the coating and substrate exposure at higher loads. Hybrid CAE-PVD/MS-PVD coatings, however, showed a more gradual failure sequence: the DLC surface layer was first abraded under increasing load, as confirmed by compositional analysis, and only after exceeding Lc did cracks from tensile stresses propagate. Although final failure was still characterised by delamination, the DLC top layer and CrN interlayer significantly modified the damage progression. The CrN layer improved interfacial bonding and reduced property mismatch between the DLC and nitride base, while the multilayer architecture promoted crack deflection and distributed stresses more evenly. Together, these factors enhanced adhesion and delayed catastrophic coating failure compared to monolithic PVD coatings.

**Figure 8:**
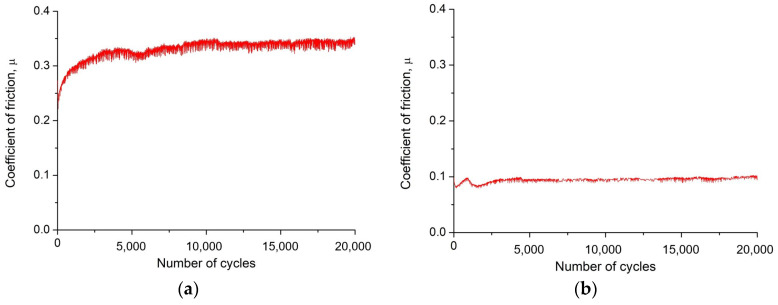
Friction coefficient as a function of the number of cycles for a coatings: (**a**) CrN, (**b**) CrN/DLC.

Based on abrasion resistance tests at ambient temperature using a ball-on-disc tribological test in reciprocating mode, recording changes in the friction coefficient µ as a function of the number of cycles, it was found that CAE-PVD coatings and, above all, CAE-PVD/DLC hybrid coatings reduce the friction coefficient compared to the uncoated material, which was hot work tool steel, both at a load of 10 N and 20 N. Due to the presence of oxides on the surface of the test steel, the coefficient of friction for the tool steel-Al_2_O_3_ (counterexample) friction pair ranged from 0.55 to 0.65 at the initial stage of the test. After the oxide layer, the coefficient of friction stabilised at 0.75 for the 10 N load and 0.90 for the 20 N load. The friction coefficient for the CAE-PVD coatings ranged from 0.35 to 0.50 for a load of 10 N (Figure 8a) and increased from 0.40 to 0.60 for a load force. The application of a DLC-type lubricating layer on the substrate material and on CAE-PVD coatings significantly reduced the friction coefficient to 0.10 for a load of 10 N (Figure 8b) and 0.14 for a load of 20 N. In the case of applying the DLC coating, a graphitisation effect was observed, causing the friction coefficient value to increase by an average of 0.05 in the first phase of the test and then stabilise at a lower constant level. As the authors demonstrated in papers [27,28,29], during abrasion of the DLC coating against the counterexample material, there is a structural transformation of the part of the coating material from an ordered diamond-shaped structure (sp^3^) to a softer and easier-to-glide graphitic structure (sp^2^). Under both dry and lubricated conditions (with a DLC layer), an increase in the coefficient of friction was observed, similar to the study in [30,31,32]. A high load leads to a higher micropitting of the material and a higher tendency to abrade, resulting in a higher friction—without the DLC layer, an increase in the friction coefficient of 0.15 occurs. In the case of lubrication, an increase in load also leads to an increase in friction coefficient, but to a lesser extent (by 0.04), due to the presence of the DLC lubricating layer, which reduces direct surface contact (Table 4).

Based on the abrasion profiles in the cross section, an increase in load also has a strong effect on the wear intensity of the tested coating systems, as a higher load leads to higher pressure on the surface, resulting in faster abrasion and damage to the surface of the material (Table 5). The wear volume for the steel substrate was 0.064 mm^3^ for the contact force of 10 N and 0.120 mm^3^ for the contact force of 20 N, respectively. When CAE-PVD coatings without a DLC layer were applied to this substrate, smaller wear volume values (up to a maximum of 0.055 mm^3^) were demonstrated. At lower loads, most wear profiles were unmeasurable. The AlCrTiN and CrN coatings wore the least under these dry conditions. At a load of 10 N, their profiles were unmeasurable, while for a load of 20 N, the wear volume was 0.005 mm^3^ for AlCrTiN and 0.008 mm^3^ for CrN. Under lubricated conditions with a DLC layer, the load also affected wear, but with lubrication, the abrasion process is more controlled and the wear is less than in dry conditions without the DLC layer. Even with higher contact forces, lubrication reduces direct surface contact, thus reducing the intensity of wear. In the cases analysed, the DLC layers applied to AlCrTiN, TiAlCN, and CrN coatings showed the lowest wear volume. The results of the aforementioned studies confirmed [24,33] that chromium as a transition layer and/or as a component of the PVD coating affects the wear resistance of DLC mainly indirectly, by reducing stress, improving adhesion, and structural stabilisation. Consequently, DLC correlated with the chromium interlayer is less susceptible to microcracking and spalling and thus more durable under abrasive conditions, which was also confirmed by further damage analysis by Scanning Electron Microscopy (SEM).

**Figure 9:**
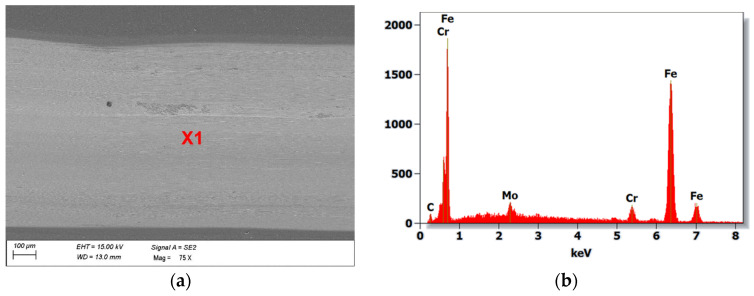
(**a**) The wear trace on the AlTiCN coating after ball-on-disc test (20 N load), (**b**) X-ray energy dispersion plot for area X1.

**Figure 10:**
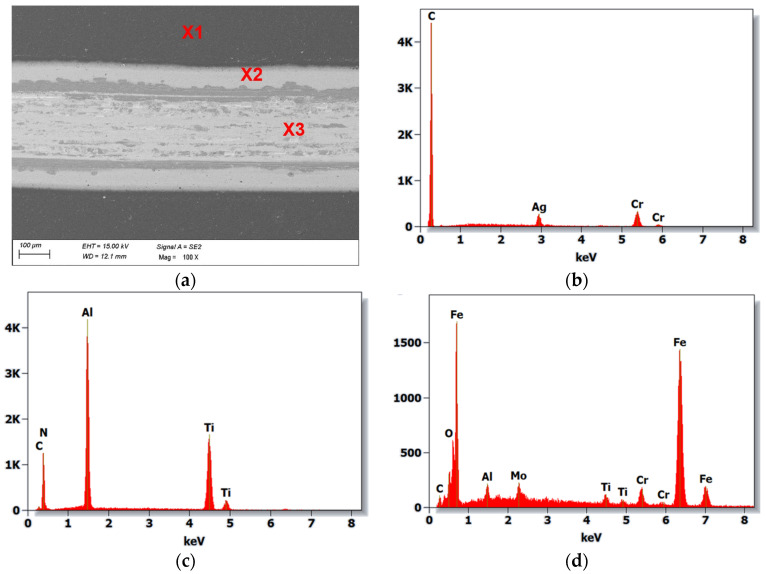
(**a**) The wear trace on the AlTiCN/DLC coating after ball-on-disc test (20 N load), (**b**) X-ray energy dispersion plot for area X1, (**c**) X-ray energy dispersion plot for area X2, (**d**) X-ray energy dispersion plot for area X3.

SEM (scanning electron microscopy) studies showed that the main wear mechanism during the ball-on-disc test of the analysed hot-work steel and CAE-PVD coatings without and with a DLC layer was abrasive. The greatest degradation occurred in the AlTiCN coating, for which the abrasion of the substrate material occurred at a load of 10 N and 20 N (Figure 9), which was confirmed by analysis of the chemical composition of the EDS. The application of a DLC layer on this coating delayed this degradation process. For a pressure force of 10 N, only the DLC layer was rubbed off, while for a pressure force of 20 N, complete abrasion of the AlTiCN/DLC hybrid coating into the substrate material was observed. In exposed areas of the substrate material, oxidised areas of the test steel were formed. Furthermore, two types of abrasive wear mechanisms can be observed for both the AlTiCN coating and the AlTiCN/DLC coating (Figure 10), which dominated during the ball-on-disc test used. The first was an adhesive mechanism characterised by the fact that the coating and friction material tended to form atomic bonds that led to the detachment of the coating fragments. This mechanism was also confirmed by analysis of the surface of the counterexample material. The second mechanism that occurred was the abrasion mechanism; in turn, in this mechanism hard particles of the abraded coating remove material from the surface by micro-cutting or microplastic deformation. In the other cases of the coated systems examined, no abrasion of the coating was observed. Instead, these coatings experience surface smoothing and removal of the highest irregularities associated with the characteristic surface topography of CAE-PVD coatings. Wear progresses at a steady rate, associated only with microstructural damage/microcracking.

**Figure 11:**
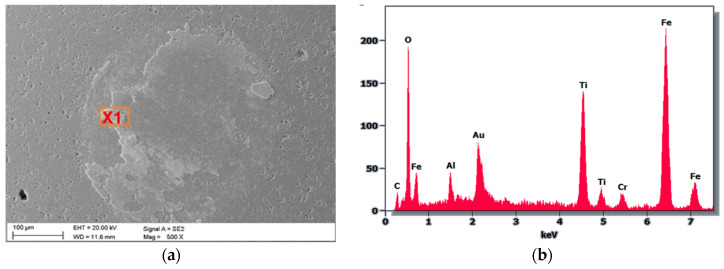
(**a**) View of Al_2_O_3_ ball after the “ball-on-disc” (20 N load) used in the TiN coating test, (**b**) X-ray energy dispersion plot for area X1.

As a result of the SEM examination of the counterexample material after the ball-on-disc test, the porous structure of the ball made of Al_2_O_3_ was observed, which is a direct result of its manufacturing technology (powder metallurgy). In addition, in the central part of the ball, the pore filling and adhesion of the friction couple material wiped off during the test are visible. Analysis of chemical composition using EDS showed that in addition to the elements present in the counterexample, the wiped coating and the substrate material of the hot-work steel are present on the surface (Figure 11). The golden reflection comes from the sputtering of the sample for SEM testing.

A ball-on-disc tribological study showed that the use of CAE-PVD coatings, especially their hybrids with a DLC layer, significantly reduces the coefficient of friction and wear compared to that of uncoated tool steel. The best sliding properties and wear resistance were achieved with DLC coatings, which stabilised friction at a low level (0.10–0.14) due to the graphitisation effect. The higher load increased wear, but the DLC coating effectively reduced the intensity of abrasion. Detailed studies by SEM together with EDS confirmed that the main wear mechanism was abrasive and adhesive degradation, and the best durability was shown by chromium and DLC coatings, which counteracted microcracking and abrasion.

Based on the measured values of the volumetric wear rate for applied coating systems subjected to abrasion tests at a load of 20 N, it was possible to quantify their wear resistance (Table 6). In the studied group of materials, the highest wear resistance was observed for multilayer coatings containing a DLC layer, such as AlCrTiN/DLC, TiAlCN/DLC, and CrN/DLC. In the cases of these coatings, values of the tested index of 0.17 ×10^−5^ mm^3^/Nm or less were recorded and, for some of them, volumetric wear was not measurable, indicating extremely high wear resistance, bordering on the absence of effective material loss under test conditions. Another group consisted of coatings such as AlCrTiN and TiN/DLC, achieving “wear rates” in the range of 0.21 to 0.33 × 10^−5^ mm^3^/Nm. Despite the absence of a DLC layer in some cases (e.g., AlCrTiN), high wear resistance was maintained, which can be attributed to the favourable mechanical and adhesive properties of these coatings. A medium level of wear resistance was represented by AlTiCN and DLC coatings. Although they are significantly more resistant than the base material, their tribological properties are clearly inferior to those of hybrid coatings. The worst resistance to wear was demonstrated by the base material without coating, wear rate = 5 × 10^−5^, which is approximately 30 times higher than in the best coatings. The above results clearly indicate that the greatest effectiveness in minimising tribological wear is provided by hybrid multilayer coatings with a DLC layer, especially in configurations with hard ceramic base layers.

An analysis of the correlation between microhardness and wear resistance indicates that the relationship is not linear and that the tribological properties of coatings are the product of both hardness and their structure, chemical composition, and surface layer characteristics. For example, the AlCrTiN/DLC coating achieved zero wear rate at a relatively low microhardness (1628 HV), which clearly indicates very high wear resistance, probably due to the synergy between the microhardness of the core and the low friction coefficient of the DLC layer. In comparison, the hardest AlTiCN coating (4220 HV) showed the highest wear rate among the tested coatings (2.29 × 10^−5^ mm^3^/Nm), suggesting that high microhardness does not automatically translate into better wear resistance, probably due to brittleness of the material or microcracking under high load. Furthermore, coatings with moderate microhardness, such as TiAlCN/DLC and CrN/DLC, showed very low wear rates (0.17 × 10^−5^ mm^3^/Nm), even though their microhardness was relatively low (1438–1567 HV), indicating the effectiveness of DLC layers as a wear-reducing component. In comparison, the uncoated substrate showed the worst wear resistance (5.00 × 10^−5^ mm^3^/Nm) at hardness 1702 HV, confirming the importance of both the selection of the coating and its overall hybrid system. In light of the above results and the literature [27], it can be concluded that high microhardness does not always correlate with low wear, and the tribological properties, adhesion, and surface layer structure are also critical.

On the basis of abrasion resistance and adhesion tests, it can be concluded that although high Lc often indicates good adhesion, it does not always correlate directly with a low wear rate. The AlCrTiN/DLC coating shows a zero wear rate and high adhesion (Lc = 105 N), making it the most sustainable protective system. The TiAlCN/DLC and CrN/DLC coatings also show very low wear (0.17 × 10^−5^ mm^3^/Nm) with high Lc (102 and 100 N, respectively), indicating the synergistic effect of the hard sublayer and the DLC coating. On the other hand, they reach the highest critical load values of Lc (120–127 N), but their resistance to abrasive wear is much lower (0.67–0.83 × 10^−5^ mm^3^/Nm). This means that despite the very good adhesion, the tribological mechanisms lead to more intense wear, probably due to brittleness or lack of self-lubrication effect. The AlTiCN coating and pure DLC show the highest wear rate (2.29 × 10^−5^ and 1.67 × 10^−5^ mm^3^/Nm, respectively) and the lowest Lc (80 N and 76 N). This suggests a limited protective effectiveness of these coatings in high-friction applications without adequate structural support.

The juxtaposition of the ‘wear rate’ and Lc indicates that the best tribomechanical properties are achieved by hybrid coating systems, in which a DLC layer is deposited on a suitable selected nitride or carbide-nitride sublayer. The AlCrTiN/DLC coating stands out as an optimal configuration, combining ideal wear resistance (wear rate = 0) with good adhesion (Lc = 105 N), making it suitable for use under extreme load-wear conditions. However, high adhesion does not always guarantee low wear; coatings such as TiAlCN or TiN exhibit very good adhesion but exhibit much higher wear rates. Similarly, the mere presence of a DLC layer is not enough—without a properly selected base layer (as in the case of a pure DLC layer), the coating’s performance characteristics are significantly reduced. In summary, the best results are obtained by synergising the mechanical properties of hard base layers such as CrN and AlCrTiN with a low-friction and wear-resistant DLC layer, highlighting the importance of designing hybrid coatings with an optimised functional layout.

## 5. Conclusions

1. The results obtained prove that hybrid coatings, in which the DLC layer interacts with a suitably selected base layer, exhibit the best tribological properties. The AlCr-TiN/DLC hybrid coating proved to be particularly effective, combining zero wear with high adhesion, making it suitable for applications under extreme load and friction conditions.
2. Contrary to initial assumptions, the coating with the highest microhardness (AlTiCN, 4220 HV) showed the highest wear, suggesting that excessive hardness can lead to brittleness and microcracks. Therefore, not only hardness but also chemical composition, adhesion, and surface layer structure play a key role.
3. A high critical load value (Lc), which indicates good coating adhesion, does not always correlate with low wear. The examples of TiAlCN and TiN coatings show that, despite high adhesion, the absence of a DLC lubricating layer or its suboptimal integration leads to more intense wear under friction conditions.
4. The mere presence of a DLC layer does not guarantee high wear resistance. In the case of DLC coatings without a properly selected layer, a significant deterioration in protective properties is observed, confirming the need to design coating systems as multilayer systems with matching mechanical and structural properties.

## Figures and Tables

**Table 1:** Chemical composition of used steel.

Element	C	Si	Mn	Cr	Mo	V
Content [%]	0.53	0.2	0.19	4.37	1.9	0.68

**Table 2:** Coatings for testing.

PVD Process Type	Coating Type	Thickness, µm
CAE-PVD	TiAlCN	4.2
	AlTiCN	2.2
	AlCrTiN	4.4
	TiN	2.8
	CrN	3.3
CAE-PVD/MS-PVD	TiAlCN/DLC	6.5
	AlTiCN/DLC	4.7
	AlCrTiN/DLC	6.5
	TiN/DLC	5.1
	CrN/DLC	5.5
MS-PVD	DLC	2.2

**Table 3:** Critical load values L_c_, N.

Coating	Critical Load L_c_, N
TiAlCN	127
TiAlCN/DLC	102
AlTiCN	80
AlTiCN/DLC	85
AlCrTiN	120
AlCrTiN/DLC	105
TiN	126
TiN/DLC	124
CrN	85
CrN/DLC	100

**Table 4:** Values of the coefficient of friction depending on the load.

Type of Coating	Coefficient of Friction µ	
	Load 10 N	Load 20 N
Substrate uncoated	0.75	0.90
DLC	0.10	0.14
TiAlCN	0.40	0.45
TiAlCN/DLC	0.10	0.14
AlTiCN	0.40	0.60
AlTiCN/DLC	0.10	0.14
AlCrTiN	0.50	0.60
AlCrTiN/DLC	0.10	0.14
TiN	0.45	0.50
TiN/DlC	0.10	0.14
CrN	0.35	0.40
CrN/DLC	0.10	0.14

**Table 5:** Wear volume in relation to load.

Type of Coating	Consumption Volume, mm^3^	
	Load 10 N	Load 20 N
Substrate	0.060	0.120
DLC	0.020	0.040
TiAlCN	0.008	0.016
TiAlCN/DLC	unmeasurable	0.004
AlTiCN	0.037	0.055
AlTiCN/DLC	0.003	0.010
AlCrTiN	unmeasurable	0.005
AlCrTiN/DLC	unmeasurable	unmeasurable
TiN	0.007	0.020
TiN/DlC	unmeasurable	0.008
CrN	unmeasurable	0.008
CrN/DLC	unmeasurable	0.004

**Table 6:** Ranking of abrasion resistance of the coating systems under 20 N load.

Type of Coating	Wear Rate [10^−5^× mm^3^/Nm]	Abrasion Resistance
AlCrTiN/DLC	immeasurable	Best
TiAlCN/DLC	0.17	Very high
CrN/DLC	0.17	Very high
AlCrTiN	0.21	Very high
AlTiCN/DLC	0.42	Very good
TiN/DLC	0.33	Very good
CrN	0.33	Very good
TiAlCN	0.67	Good
TiN	0.83	Good
AlTiCN	2.29	Average
DLC	1.67	Average
Substrate uncoated	5.00	Poor

## Data Availability

The original contributions presented in this study are included in the article. Further inquiries can be directed to the corresponding authors.
